# Ginkgo Biloba and Green Tea Polyphenols Captured into Collagen–Lipid Nanocarriers: A Promising Synergistically Approach for Apoptosis Activation and Tumoral Cell Cycle Arrest

**DOI:** 10.3390/ijms26199648

**Published:** 2025-10-03

**Authors:** Mirela Mihaila, Nicoleta Badea, Marionela Birliga, Marinela Bostan, Madalina Georgiana Albu Kaya, Ioana Lacatusu

**Affiliations:** 1 Stefan S. Nicolau Institute of Virology, Mihai Bravu Street No 285, 030304 Bucharest, Romania; mirela.mihaila@virology.ro (M.M.); marinela.bostan@virology.ro (M.B.); 2 Faculty of Pharmacy, Titu Maiorescu University, Bd. Gh. Sincai No. 16, 040314 Bucharest, Romania; 3 Faculty of Chemical Engineering and Biotechnologies, National University of Science and Technology Politehnica Bucharest, Polizu No 1, 011061 Bucharest, Romania; nicoleta.badea@upb.ro (N.B.); marionela.birliga@stud.chimie.upb.ro (M.B.); 4 Department of Immunology, Victor Babes National Institute of Pathology, 99-101 Splaiul Independetei, 050096 Bucharest, Romania; 5 Division of Leather and Footwear Research Institute, Department of Collagen, National Research and Development Institute for Textiles and Leather, 93 Ion Minulescu Street, 031215 Bucharest, Romania; albu_mada@yahoo.com

**Keywords:** Collagen-nanostructured lipid carriers, Ginkgo Biloba entrapment, hepatocyte tumor cells apoptosis, phytochemicals cell cycle arrest

## Abstract

Considering the world’s growing interest in health-promoting phytochemicals, the current research investigated the development of a dual-captured *Ginkgo Biloba* and Green Tea Extract into Collagen-Nanostructured Lipid Nanocarriers (Col-NLC-*GBil*-GTE) for an enhanced therapeutic efficacy against hepatic, colon or breast cancer. NLC considerably reduced cell viability; the most advanced cytotoxicity profile was determined on human colon adenocarcinoma cells (LoVo) and liver cancer cells (HepG2), e.g., tumor cell viability was 21.81% in the presence of Col-NLC-*GBil*-GTE, similar to that determined for Cisplatin. Col-NLC exhibited apoptosis in HepG2 and LoVo cells and no significant apoptosis induction in normal HUVECs. A 20% increase in apoptosis for HepG2 cells was registered for 100 μg/mL NLC-GBil-GTE compared to Cisplatin (Cis-Pt), e.g., a 63.4% total apoptosis for NLC-*GBil*-GTE versus a 52.6 apoptosis induced by 100 μg/mL of a chemotherapeutic drug. According to the cell cycle outcomes, an accumulation of hepatocyte HepG2 tumor cells in the G0/G1 phase was detected upon treatment with 100 mg/mL of NLC- and Col-NLC-*GBil*-GTE, simultaneously with a drastic decrease in the S phase, which may indicate a cell number reduction that enters in the division cycle. The simultaneous delivery of GBil and GTE by synchronizing their bioactivities offers several advantages; Col-NLC-*GBil*-GTE can be viewed as a noteworthy strategy for consideration in connection with antitumor therapeutic protocols.

## 1. Introduction

Cancer, a global health issue and the greatest enigma of human health nowadays, spreads, in general, because the largely changing lifestyle and an ever-increasing rise in carcinogenic substances has caused an alarming increase in statistics every year [1]. One of the go-to approaches to increase therapeutic efficacy is the use of nanocarriers to co-deliver the active principles alongside potentialities to sensitize cancerous cells or improve therapy efficacy [2,3]. Recent advances in lipid-based nanocarriers demonstrated the interest and considerable attention of scientists to natural products [4] and their potential for enhancing therapeutic efficacy [5].

**Figure 1:**
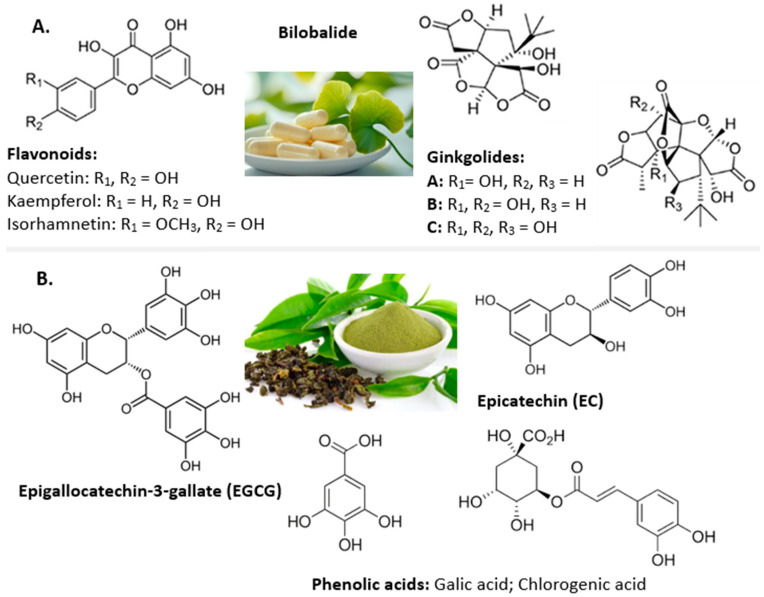
Key bioactive phytochemicals present in *Ginkgo biloba* extract (**A**) and green tea extract (**B**).

Nanostructured lipid carriers (NLCs) are promising delivery systems for anticancer agents, including natural extracts, due to their ability to improve the solubility, stability and bioavailability of phytocompounds [6], as well as to facilitate the targeting of tumor cells [7]. They incorporate a mixture of solid and liquid lipids (oils) in their matrix, creating a disordered structure that is therefore capable of loading more active substances and ensuring a more efficient release [8]. NLCs have emerged as a unique delivery system because of their benefits, such as their easy preparation techniques for large-scale production [9], low toxicity [10] and high drug loading capacity [11], and, most importantly, NLCs are very suitable for co-delivery in cancer treatment [12]. NLCs are suitable for improving cellular targeting and reducing the systemic toxicity of bioactive compounds, including *Ginkgo Biloba* extract (*GBil*). *GBil* leaf extract has a long history in traditional medicine for use as a complex source of biologically active phytocompounds [13]. *GBil* extract is rich in bioactive compounds, especially flavonoids (e.g., quercetin, kaempferol, isorhamnetin, bilobetine), phenolic acids (chinic acid, shikimic acid, gallic acid, vanilic acid, etc.) and terpenoids (ginkgolides and bilobalides) [14]. Some of the major polyphenolic phytocompounds in the extract, as well as various Ginkgolide-type terpenoids, are presented in Figure 1. *GBil* possesses valuable therapeutic properties, particularly in the neurological, cardiovascular and antitumor fields [15,16]. It has been shown to have a variety of medicinal and pharmacological properties, including antidementia, anticancer, antimicrobial, antioxidant, antilipidemic, antidiabetic, antiobesity, antiplatelet, anti-inflammatory, hepatoprotective, antidepressant, antihypertensive and neuroprotective effects [14,17]. *GBil* extracts have demonstrated the ability to inhibit proliferation in breast cancer cell lines, MCF-7 and MDA-MB-231 [18], and to induce differentiation and apoptosis in cervical cancer cells [19] and in colorectal cancer cell lines [20]. They can suppress the growth of ovarian cancer cells by inducing apoptosis (increasing the expression of pro-apoptotic proteins, Bax, and decreasing the anti-apoptotic ones, Bcl-2) and by inhibiting angiogenesis [21]. Certain components of *GBil* have been investigated for their potential to inhibit the growth of glioblastoma (brain cancer) cells [22].

More than 50 flavons were identified in *GBil*, and the components with the highest response biological values were Quercetin, Kaemperol, Isorhamnetin, Neoisorutin, Hyperoside, Amentoflavone, Bilobetin, Ginkgetin and Isoginkgetin [23]. Flavonoids have demonstrated the potential to regulate metabolism and proteins/enzymes related to tumor cell growth and survival, with numerous studies highlighting their relationship with tumor growth and cancer metastasis associated with angiogenesis [24]. Quercetin, Kaempferol and Isorhamnetin act as powerful antioxidants, neutralizing free radicals (which damage DNA, lipids and proteins) and inhibiting the initiation of cancer cell formation. They inhibit tumor growth through mechanisms such as blocking the cell signaling pathways involved in proliferation and metastasis, inducing apoptosis and inhibiting angiogenesis [25]. According to Menezes et al., biflavonoids from *GBil* can exert therapeutic benefits by regulating several proteins/enzymes (PPAR-γ, CCAAT/enhancer-binding protein α, STAT5, pancreatic lipase, PTP1B, fatty acid synthase, α-glucosidase) and insulin signaling pathways (via PI3K-AKT), which are linked to metabolism, cell growth and cell survival mechanisms [24]. The translational role of biflavones in cancer with respect to the inhibition of metabolism-related pathways, enzymes or proteins (such as STAT3/SHP-1/PTEN, kinesins, tissue kallikreins, aromatase, estrogen, protein modifiers), and antioxidant, autophagy and apoptosis induction mechanisms are discussed in several studies [23,24,25]. Ginkgetin was found to inhibit the phosphorylation of STAT3, JAK1 and c-Src kinases in a dose-dependent manner in A549 lung carcinoma and hypopharyngeal carcinoma cells. The activated signal transducer and activator of transcription 3 (STAT3) plays an effective role in normal cell survival and apoptosis in human cancer cells. Terpenoids (Ginkgolide and Bilobalide) have a different but complementary mechanism of action to flavonoids. In vitro and in vivo studies have demonstrated an ability to inhibit cancer cell growth and induce apoptosis [26].

However, *GBil* has some limitations associated with its low bioavailability and rapid degradation along the gastrointestinal tract. To overcome the challenges related to the low oral bioavailability of certain phytocompounds from *GBil,* lipid-based delivery systems such as Solid Lipid Nanoparticles/SLNs [27] and nanostructured lipid carriers/NLCs [28] have been investigated. The aim was to improve bioavailability, allowing for the controlled release of bioactive phytocompounds [29], to optimize toxicity [30], to enhance antioxidant and anti-inflammatory activities [31,32] and to ensure neuroprotective and antitumor efficacy [33,34].

Another natural polyphenolic mixture widely studied due to its extensive biological effects is *Camellia sinensis*, known as green tea extract (GTE). GTE exhibits biological and pharmacological properties such as anti-inflammatory, antimicrobial, antiviral, and anticancer activities [35]. The health-related effects, and especially the significant antitumor potential, of GTE are mainly attributed to the bioactive polyphenolic compounds, for example, catechins such as epigallocatechin gallate (EGCG), epicatechin gallate (ECG), epigallocatechin (EGC) and epicatechin (EC) [36]. GTE phytocompounds, particularly EGCG, can induce cytotoxicity in a wide range of tumor cell lines. GTE can block cell cycle progression in different phases, G0/G1 or G2/M [37], and can initiate intrinsic and extrinsic apoptotic pathways in tumor cells [38], involving the activation of caspases, damage to the mitochondrial membrane and the release of cytochrome c [39,40], ultimately leading to programmed cell death (apoptosis). GTE also has anticancer effects by playing an essential role in inhibiting the epidermal growth factor receptor (EGFR) angiogenesis marker [41] or inhibiting tumor cell invasion and metastasis by modulating the activity of the metalloproteinases involved in the degradation of the extracellular matrix [42]. Like most natural products, its application is limited due to its low stability and low oral bioavailability [43]. Therefore, nanoencapsulation is a strategic option to fully exploit its therapeutic potential. Several studies have investigated the improved stability and bioavailability of tea polyphenols loaded into SLNs [44] and NLCs [45], and their suitability for the treatment of breast cancer through in vitro and in vivo studies [46].

The combinatorial delivery of *GBil* and GTE, mediated by NLCs and Collagen–NLCs, represents a promising strategy to improve the stability (mainly of pH and along the gastrointestinal tract), bioavailability and, implicitly, antioxidant and antitumor efficacy of these highly therapeutically valuable phytochemical mixtures. The simultaneous delivery of *GBil* and GTE by synchronizing their bioactivities offers several advantages, including the following: *(i).* the protection of polyphenols from enzymatic and chemical degradation, maintaining pharmacological integrity over a longer period; *(ii).* an improved absorption through cell membranes and a more efficient delivery of phytochemicals in tissues; and *(iii).* a sustained controlled release of bioactives from the NLC, maintaining a constant therapeutic phytoconstituent concentration. Furthermore, the NLC surface can be modified for the specific targeting of tumor cells, thus increasing the effectiveness of delivering the natural bioactive. In this study, for the first time, we used NLC and collagen-decorated NLC for a combinatorial co-delivery of *Ginkgo biloba* alongside the chemotherapeutic mixture of green tea polyphenols with the aim to intensify cancerous cells’ sensitization, to display ideal synergistic cytotoxic effects against cancer cells and to improve therapeutic efficiency. Thus, the current research investigated the prominent potential of individual and dual phytochemical-loaded NLCs and Collagen–NLCs of GTE and *GBil* to augment their anticancer effects against colon, hepatic and breast cancer cells and to open a new horizon in the field of cancer therapy.

The importance of coating a lipid delivery system with hydrolyzed collagen is fundamental because it provides the premise for the development of safe, efficient and targeted nanocarrier systems, with a major impact in enhancing therapeutic efficacy in hepatic, colon or breast cancer. As a natural tissue polymer, collagen has an improved safety, biocompatibility and biodegradability, and thus minimizes the risk of toxicity and allows nanocarriers to be metabolized and safely eliminated from the body after targeted drug delivery to specific cells [47]. Due to specific peptide sequences that are recognized by cell surface receptors, collagen exhibits an improved cell adhesion and tissue integration [48]. Laghezza et al. showed that coating nanocarriers with collagen improved the surface adhesion of nanoparticles to the target cells and facilitated their internalization [49]. Decoration with collagen also confers physical and structural protection to nanoparticles, increasing their stability in the physiological environment [50]; for instance, it can improve resistance to phospholipase activity and reduce phagocytosis by the reticuloendothelial system. Furthermore, collagen can prevent nanoparticle aggregation, the degradation of lipid components, and the premature release of active principles [51].

## 2. Results and Discussion

For the preparation of the NLC, a lipid matrix consisting of sesame oil, cocoa butter and glyceryl monostearate was used. Sesame oil includes mostly unsaturated fatty acids, but also other important bioactive compounds in terms of antioxidant and antitumor potential [52]. Cocoa butter, also used to define a suitable solid lipid core, has beneficial properties for health, such as reducing inflammation, improving blood vessel elasticity and lowering blood pressure [53]. To stabilize the core and facilitate weak interactions such as hydrogen bonds, electrostatic attractions with hydrolyzed collagen molecules, soy lecithin and an alkyl sorbitan (Tween 20) were used.

### 2.1. Evaluation of the Size and Effectiveness of Individual and Mixed Capture of GBil and GTE + GBil in Various NLCs and Col-NLCs

**Figure 2:**
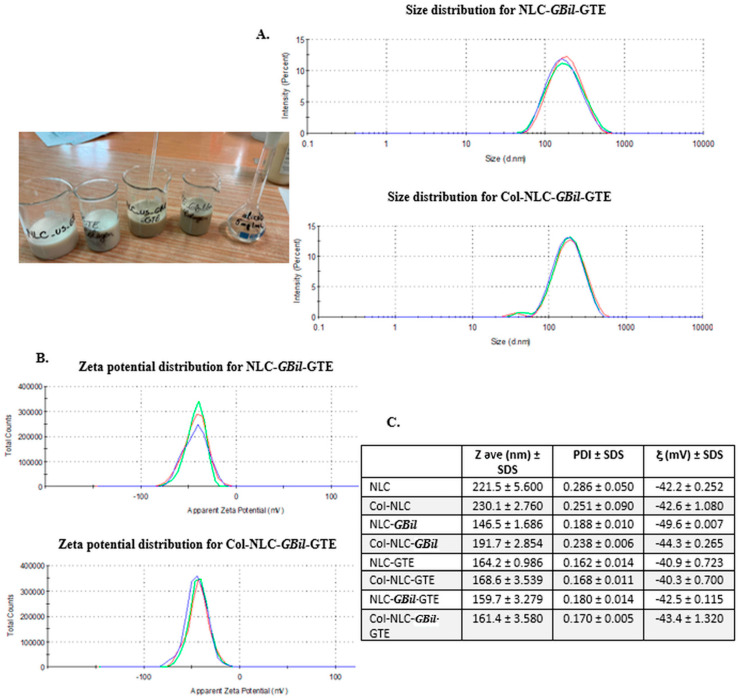
Size distribution curves (**A**) and zeta potentials (**B**) of NLC- and Col-NLC. Zave, PdI and ξ values evaluated by DLS analysis (**C**).

The size characterization for NLC and Col-NLC formulations reported a narrow size distribution and negative zeta potentials. NLC and collagen-decorated NLC loaded with individual and mixed *GBil* and GTE showed a monomodal size distribution, with an appropriate homogeneity and uniform distribution of the lipid population (Figure 2A). The distribution curves are relatively narrow, a sign of a low polydispersity index, which confirms the colloidal stability and quality of the obtained nanocarriers. NLCs and those coated with collagen, which capture the active principles (*GBil* and GTE), presented average diameters, Zave < 190 nm, compared to the delivery nanocarrier without phytochemical content. This result reveals a reorganization at the surface of the lipid and lipid–peptide nanocarriers, simultaneously with the existence of the potential emulsifier effect of GBil/GTE. NLC systems with green tea and with a mixed blend of *GBil* and the GTE ones presented the narrowest size distribution, with PdI < 0.18. Coating NLCs with collagen led to a slight increase in particle size (Figure 2C) due to the adhesion of the protein layer to the NLC surface, and by electrostatic interactions and H-bonds formed between surfactants and peptide chains. The size distribution remained relatively narrow also in the case of Col-NLC-*GBil*-GTE, which shows that the stability of the distribution of phytochemicals was preserved.

The surface charge of the NLC and Col-NLC was comparatively evaluated (by the specific parameter, zeta potential) as an indicator of the physical and chemical stability of the prepared nanodelivery systems. Our colloidal nanocarriers have a negative surface charge, which creates repulsive forces that prevent the particles from adhering. For the stabilization of colloidal systems, a minimum zeta potential of ± 30 mV is required for the repelling of particles from each other [54]. Although the formulations with nonionic surfactants do not confer low zeta potential values, the stability of the system is conferred by the steric barrier generated by the complex structure of alkyl sorbitans [55]. As may be seen from Figure 2C, all formulations presented potential values favorable for the appropriate colloidal stability, ξ < −50 mV, which means avoiding coalescence and flocculation phenomena.

The entrapment efficiency of polyphenols from *GBil* and/or GTE in the NLC and collagen-coated NLC formulations was determined by the Folin–Ciocalteau spectrophotometric method. The total polyphenol content was expressed in gallic acid equivalents, mg GAE/mL, and provided a clear indicator of the capacity of NLCs and Col-NLCs to efficiently capture the active phytocompounds from *GBil* and GTE. According to the obtained results (Table 1), no significant differences were reported between NLCs and lipid–protein nanocarrier systems containing phytochemical mixtures. However, it was observed that NLC formulations containing GTE showed a slightly higher entrapment efficiency (>85% polyphenols entrapment) compared to those containing only *GBil* (e.g., EE% for NLC-*GBil* of 72.30% ± 0.742 versus 86.75% ± 1.213 for NLC-GTE). These results suggest a better structural and conformational adaptability of GTE in the nanocarrier matrix, most likely due to its higher solubility and affinity for the surfactant blend used to stabilize the lipid core. The decoration of NLC-*GBil* with hydrolyzed protein insignificantly decreases the entrapment efficiency, while, in the case of NLC-GTE formulations, a more obvious decrease was determined, from 86.75% to 83.59%.

### 2.2. In Vitro Screening of Antioxidant Activity and Determination of IC_50_

**Figure 3:**
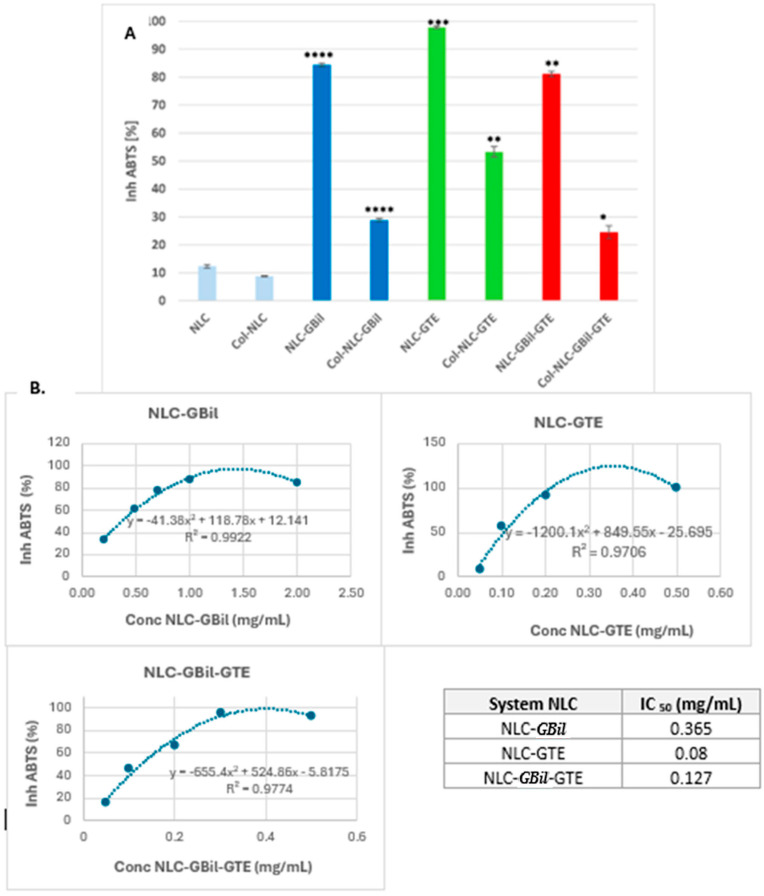
(**A**) Variation in antioxidant activity of NLC and collagen-decorated NLC phytochemicals. (**B**) ABTS⁺ radical inhibition curve as a function of NLC concentration. IC_50_ values obtained for NLC-*GBil,* NLC-GTE and NLC-*GBil*-GTE in the ABTS assay. All experiments were performed in triplicate. * *p* < 0.05; ** *p* < 0.005; *** *p* < 0.005; **** *p* < 0.0005. Data are expressed as mean ± SD, *n* = 3, NLC vs. other groups.

The plant kingdom represents an inexhaustible natural source of bioactive compounds that have multiple beneficial effects on human health. *Camellia sinensis*/GTE [56] and *Ginkgo biloba* [13] bring together a wide variety of polyphenols such as flavonoids, anthocyanins, tannins, ginkgolides and bilobalides, etc. These are powerful antioxidants that can neutralize reactive oxygen species (ROS) and reduce oxidative stress, which is often increased in cancer cells and can contribute to disease progression. For the in vitro screening of antioxidant activity, the neutralization of ABTS^+^ cationic radicals by the antioxidants present in NLCs (both lipophilic and hydrophilic in nature) was monitored. All NLC–phytochemical mixture formulations successfully fulfilled their role as strong antioxidants, showing ABTS^+^∙inhibitions greater than 80% (Figure 3). The most effective proved to be NLCs containing green tea extract, which showed an excellent ability to scavenge cationic radicals, with maximum values reaching 97.9% ± 0.43, for NLC-GTE (Figure 3A). The addition of GTE to NLC-*GBil* considerably improves the free radical neutralization capacity, most likely due to a synergistic effect between the polyphenolic compounds of the two extracts. Although NLC-*GBil* also exhibits a suitable capacity to remove free radicals, its activity was somewhat lower compared to that of NLC-GTE, 84.4% ± 0.62. The superior antioxidant activity of NLC-GTE could be related to the enriched composition of green tea epigallocatechin derivatives [56] and the nanoencapsulation effect; these results agree with the data recently reported by Yi et al. [57].

An aspect that is worth noting is the influence of the hydrolyzed collagen coating of the NLC on the antioxidant ability. According to the outcomes obtained, the antioxidant activity of NLCs was reduced when they were decorated with collagen (Figure 3A). This phenomenon is mainly due to the complex interactions between the bioactive phytocompounds from GTE and *GBil* and collagen. Both categories of extracts—*GBil* and GTE—being rich in flavonoids, epigallocatechin derivatives and terpene lactones, which contain multiple hydroxyl groups, can form strong hydrogen bonds or hydrophobic interactions with the polypeptide chains of collagen. These interactions “block” the antioxidant compounds, and, as such, they become less available to interact with free radicals. The interactions between polyphenols and protein and their influence on antioxidant activity have been reported in the literature [58,59]. In addition, the polyphenols may undergo spatial structural changes due to interactions with peptides, reducing their ability to donate electrons or protons, which is essential for neutralizing reactive oxygen species (ROS). Our results agree with those reported in related studies. For example, Sartaj et al. [46] and Feng et al. [56] reported the impact of several polymer coatings, e.g., collagen and curcumin, on the antioxidant and anti-inflammatory activity of natural actives in lipid nanoparticles.

Another defining aspect that leads to a reduction in antioxidant activity in the case of Col-NLC refers to the creation of a physical barrier concomitantly with the reduction in the phytochemical’s diffusion. The collagen layer of the NLC can act as a physical barrier, hindering the efficient diffusion of antioxidant GTE and *GBil* phytocompounds from the NLC to the external environment, where they should neutralize free radicals. As a result, collagen, although excellent for biocompatibility and targeted applications, can compromise the accessibility and efficacy of antioxidants.

For the NLCs that demonstrated the best efficacy in terms of antioxidant activity, IC_50_ values were determined (Figure 3B). It should be mentioned that the IC_50_ values included the entire lipid nanocarrier formulation, considering the potential involvement of the sesame oil and cocoa butter from the NLC’s core in combating dangerous reactive species. The IC_50_ value determined for NLC-*GBil* was 0.365 mg/mL, while NLC-GBil-GTE presented a significantly lower IC_50_ value of 0.127 mg/mL. The considerably higher antioxidant efficacy of green tea extract is indicated by the IC_50_ value of NLC-GTE, which is 0.08 mg/mL. The lower IC_50_ value reveals a higher antioxidant efficiency, suggesting an enhanced capacity to neutralize free radicals at a lower concentration of the sample. These results support the idea that the addition of green tea extract to NLC-*GBil* considerably improves the free radical neutralization capacity, most likely due to a synergistic effect between the polyphenolic compounds of the two extracts.

### 2.3. Outcomes of In Vitro Antitumoral Effect of NLC- and Col-NLC-GBil-GTE

Cell cycle analysis is a crucial technique for the screening of potential antitumor agents, providing information on how they affect cell proliferation. One of the key factors that allows tumor cells to proliferate uncontrollably is the amplification of the cell division process. Cell cycle assessment (which analyses DNA content) allows the population to study in different stages of the cell division; as such, the percentage of each cell cycle phase (G0/G1, S and G2/M) can be established, providing a useful understanding of the NLC’s mechanism. By co-opting the two categories of GBil and GTE actives in NLCs and NLCs coated with collagen, we pursued the hypothesis of their antitumor action using cell lines derived from human hepatic adenocarcinoma HepG2 (human hepatic adenocarcinoma cells), colon (LoVo) and MDA-MB 231 (human breast adenocarcinoma cells), compared to normal human umbilical vein endothelial cells (HUVECs) used as a control. The expected antitumor activity of NLC-GBil and Col-NLC-GBil with or without GTE is supported by the presence of primary flavonoids and biflavonoids, and terpene lactones in GBil. Quercetin, Kaempferol and Isorhamnetin exert their antitumor effects by targeting multiple cellular pathways and molecules, including those involved in inflammation, cell cycle regulation and tumor development [60]. The 1,3-dihydroxyphenyl structure found in the flavonoid skeleton (named “resorcinol”) is the most common feature biogenetically in several anticancer drugs and clinically tested flavones [61]. Biflavones with this resorcinol moiety have shown positive antiproliferative effects, reducing the breast cancer cells by inducing apoptosis observed via the cleavage of Caspase-3 and PARP [62]. Amentoflavones and Biapigenins are agonists of PPAR-γ and influence Nuclear Factor kappa B (NF-κB). The activation of PPAR-γ as a therapeutic target for cancer may function as a suppressor by increasing the induction and differentiation of apoptosis. NF-κB regulates Bcl-2 activity and leads to cell cycle arrest [24]. Other flavonoids from GBil, i.e., Ginkgetin, Isoginkgetin, Bilobetin, etc., reduced cell viability in a dose-dependent manner in human renal cells and human normal hepatocytes. They can exert antitumor benefits by regulating several proteins or enzymes which are linked to cell growth and cell survival mechanisms [24]. Ginkgetin has shown anticancer effects in vitro and in vivo in many cancers [63,64]. Ginkgetin causes cell cycle arrest, with an accumulation of prostate cancer cells in the G0/G1 phase of the cell cycle in a time-dependent manner. It has been reported to induce cell apoptosis in a variety of cancer cell lines, including breast cancer, colon cancer, hepatocellular carcinoma, kidney cancer, ovarian cancer and prostate cancer [24,65].

#### 2.3.1. Cell Cytotoxicity Investigations for Different NLC and Col-NLC Phytochemicals Against Various Tumor Cells

**Figure 4:**
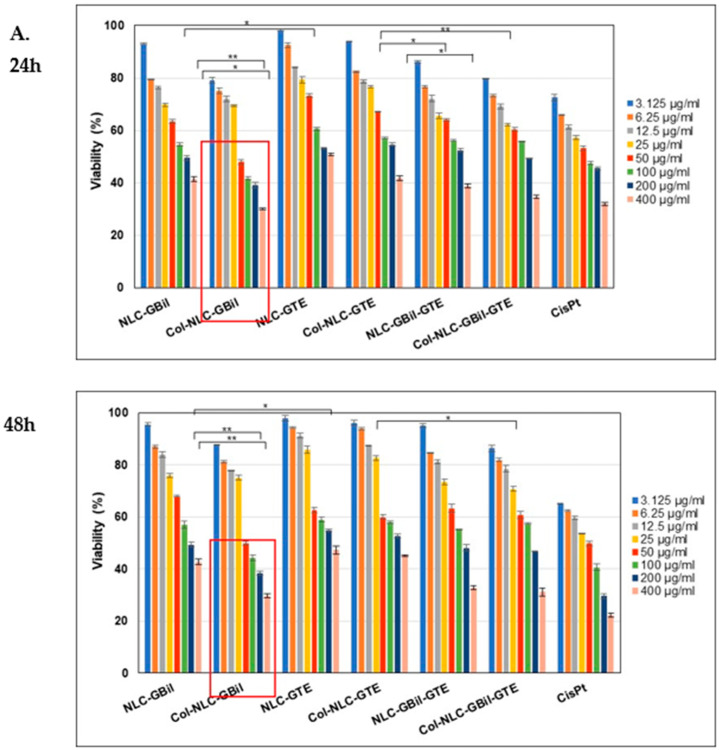

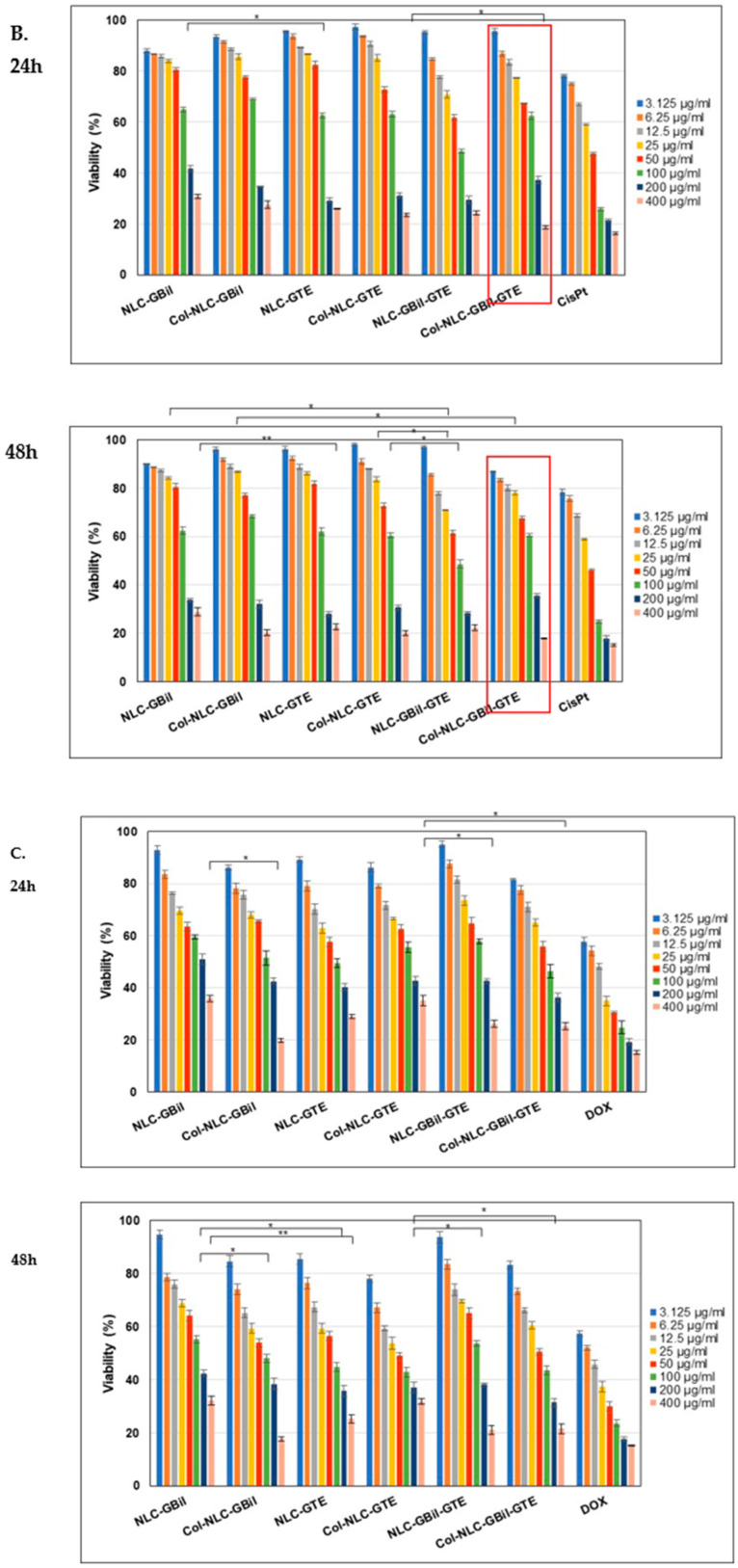

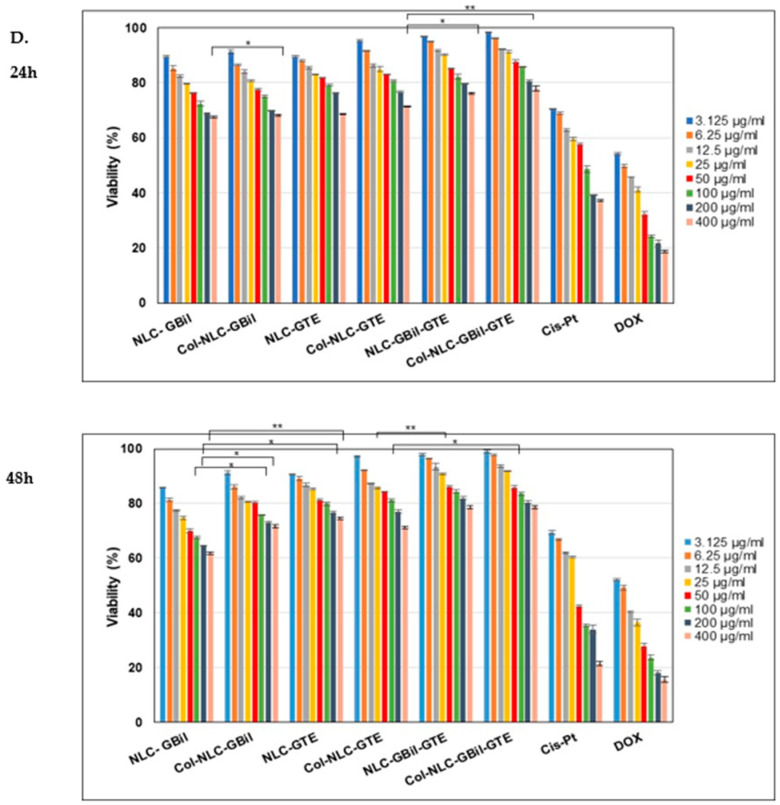
Cell viability investigations of different cells treated with different lipid nanocarriers, with and without collagen coating, for 24 and 48 h. (**A**) Human hepatic adenocarcinoma (HepG2); (**B**) human colorectal adenocarcinoma (LoVo); (**C**) human breast adenocarcinoma (MDA-MB-231); (**D**) human umbilical vein endothelial cells (HUVEC). All experiments were performed in triplicate. * *p* < 0.05; ** *p* < 0.005; data are expressed as mean ± SD, *n* = 3, NLC vs. other groups.

The incubation of tumor cells with different concentrations of NLC-*GBil*/GTE, Collagen-NLC-*GBil*/GTE and Col-NLC-*GBil*-GTE for specific treatment periods of 24 and 48 h highlighted a decrease in cell viability and, implicitly, an increased cytotoxicity manifested by the tested nanocarrier systems (Figure 4). As shown in Figure 4A, Col-NLC-*GBil* had a considerable impact on hepatic adenocarcinoma HepG2 cell viability. This formulation led to advanced cytotoxicity (loss of cell membrane integrity or denaturation of essential enzymes), reflected by a reduction in MTS absorption and a decrease in viable and functional cells, e.g., <30% cell viability (Figure 4A).

A notable aspect of the study is the comparison with reference chemotherapeutic drugs. On the LoVo cell line (colon cancer), the cytotoxic effect of the Col-NLC-*GBil*-GTE formulation was almost like that determined for Cisplatin. The similarity with Cisplatin is relevant because it suggests that NLC-active principles could represent an alternative in cancer therapy, with a better safety profile than conventional chemotherapeutics.

The in vitro evaluation of the cytotoxic potential on cancer cell viability (over a 24-h treatment period) showed a <20% decrease in the viability of colon tumor LoVo cells in the presence of Col-NLC-*GBil*-GTE. This result demonstrates that entrapping *GBil* and GTE with an NLC considerably reduces cell viability, which may be related to successful Col-NLC cellular internalization. In a related study, Wang et al. reported that *GBil*-loaded Solid Lipid Nanoparticles demonstrated an enhanced cytotoxicity and increased cellular uptake in breast cancer cell lines [34]. Other research exploring *GBil* nanoformulation for the treatment of liver cancer showed the preferential accumulation of liposomes in liver tumors and significant inhibition of tumor growth, with minimal side effects [66]. In addition to *GBil,* GTE has also demonstrated a significant antitumor potential through multiple mechanisms which directly or indirectly target the proliferation and survival of cancer cells. In agreement with our results, green tea extract induced a selective cytotoxicity on a wide range of tumor cell lines, with a minimal effect on normal cells [67]. A conclusive example reported by Zeng et al. is the efficacy of EGCG-loaded NLCs on breast cancer cell lines (MCF-7 or MDA-MB-231). EGCG-NLC demonstrated an enhanced cytotoxicity due to its improved delivery and increased cellular uptake [68].

The normal human endothelial HUVECs were used to illuminate conclusions about improving effectiveness, and the results showed a cell proliferation inhibitory effect on tumoral cells incubated with NLC and Col-NLC phytochemicals. As illustrated in Figure 4D, the reduction in HUVEC viability after exposure to NLC and Col-NLC is not as strong as cancer cells treated with those treatments over 24 h and 48 h, which likely relates to the difference in the type and the number of membrane lipids between cancer and healthy cells. As shown in Figure 4D, the effect of NLC-herbal principles and Col-NLC-herbal principles on HUVECs showed a lack of cytotoxicity, the cell viability being higher than 80% at a concentration of ≤100 mg/mL and 48 h, which suggests it is partly biocompatible.

#### 2.3.2. Apoptosis Induced by NLC- and Collagen-Decorated NLC-GBil-GTE

**Figure 5:**
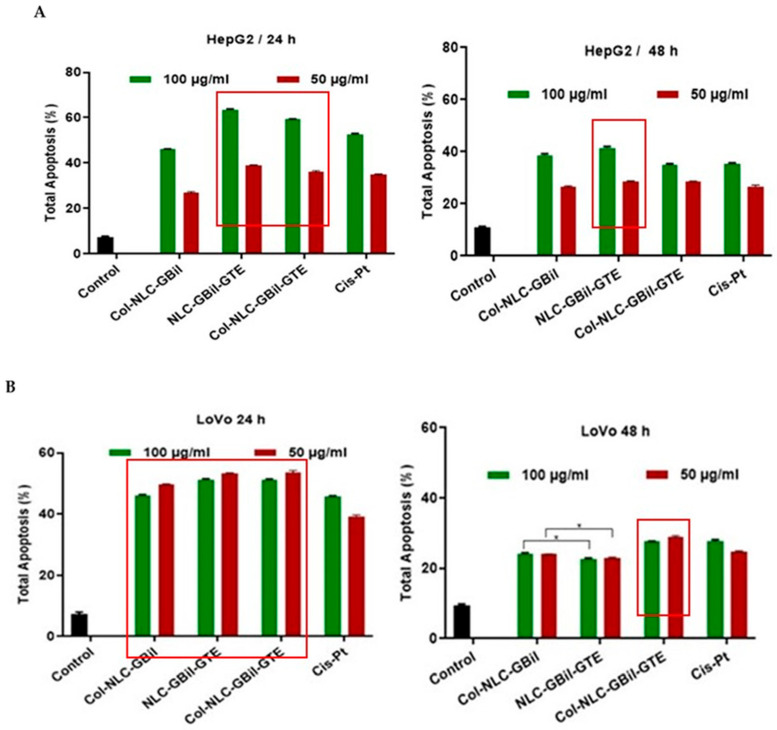

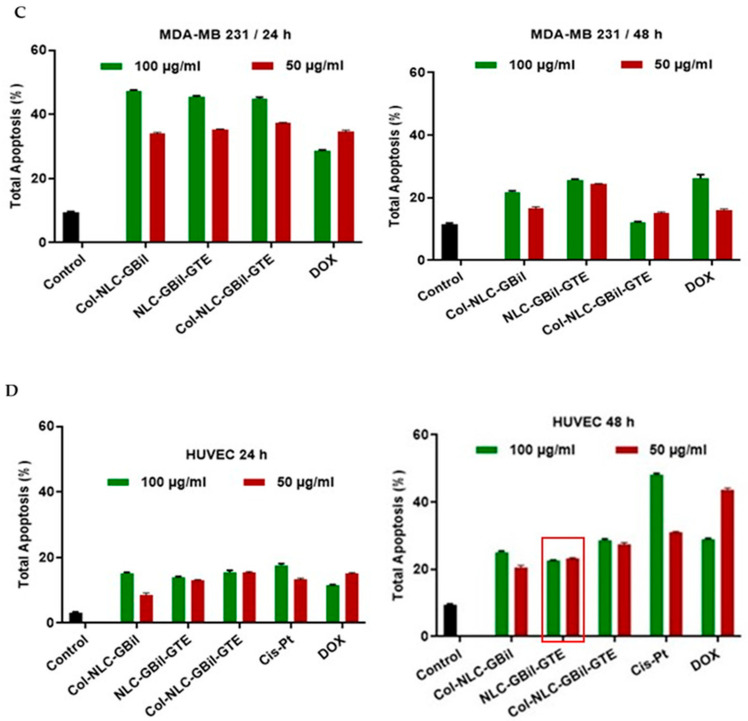
Total apoptosis induced by NLC- and Col-NLC-*GBil*-GTE on cell lines derived from human hepatic adenocarcinoma cells, HepG2 (**A**), human colorectal adenocarcinoma cells, LoVo (**B**), human breast adenocarcinoma, MDA-MB-231 (**C**), and human umbilical vein endothelial cells, HUVEC (**D**).

Understanding the mechanisms responsible for tumor formation and progression is crucial for the development of effective cancer treatments. There is various scientific evidence that continues to elucidate the complex mechanisms by which the phytocompounds contained in *GBil* and green tea extract exert antitumor effects, particularly by modulating the cell cycle and inducing apoptosis. Apoptosis is introduced as an ordered and coordinated cellular death process that happens in physiological and pathological conditions. By targeting this mechanism, the survival, growth and spread of tumors can be inhibited. Here, the developed NLCs appeared to significantly cause a 50% increase in apoptosis after their addition to tumor cells compared with the control group (Figure 5).

The results from the apoptotic investigations confirmed the early and late apoptosis of HepG2 and LoVo cells and the safety of Col-NLC, with no significant apoptosis induction in normal HUVECs. They indicated that 100 mg/mL Col-NLC coloaded with a mix of *GBil* and GTE could induce more apoptosis (around 64%) in human hepatic adenocarcinoma (HepG2) cells as compared to Col-NLC, which entrapped only *GBil* (46.1% total apoptosis). The apoptotic effect of GTE and *GBil* on cancer cells has been studied in several studies. It has been proven that polyphenolics from GTE could induce apoptosis in tumor cells by decreasing anti-apoptotic proteins, such as Bcl-2 and Bcl-xL, and by increasing the pro-apoptotic proteins, i.e., Bax and Bad [69]. Similarly to GTE, bioactives in *Ginkgo biloba* exert antitumor effects through multiple, often interconnected mechanisms that target the distinctive characteristics of cancer cells [70]. *GBil* may activate intrinsic and/or extrinsic apoptotic pathways in cancer cells. This often involves modulating the expression of pro-apoptotic proteins—Bax and Bak—and anti-apoptotic proteins—Bcl-2 and Bcl-xL [71].

Apoptosis outcomes of NLC and Col-NLC formulations underline a slightly more pronounced efficacy of NLC– and Col-NLC–phytochemical mixes compared to the usual drug, Cisplatin, at both concentrations. A 20% increase in apoptosis for HepG2 cells was registered for a treatment with a 100 μg/mL NLC–phytochemical mix, compared to Cis-Pt, e.g., 63.4% total apoptosis for NLC-*GBil*-GTE and 59% for Col-NLC-*GBil*-GTE, while the total apoptosis induced by 100 μg/mL of a chemotherapeutic drug, Cis-Pt, was 52.6% (Figure 5A).

As indicated in Figure 5B, 50 mg/mL NLC- and Col-NLC-*GBil*-GTE appeared to significantly increase, by 50%, early and late apoptosis after addition to LoVo cancer cells compared with the control group (for 24 h); for instance, NLC-*GBil*-GTE induced 53.2% apoptosis, Col-NLC-*GBil*-GTE induced 53.5% apoptosis, and 50 μg/mL Cis-Pt resulted in 39.3% apoptosis. The collagen coating of the NLC did not produce major changes at 24 h of treatment; instead, a slight enhancement of LoVo cell apoptosis was observed after 48 h, upon treatment with 50 mg/mL Col-NLC-*GBil*-GTE (Figure 5B).

Our results showed that treating the MDA-MB-231 breast tumor cells with 100 mg/mL Col-NLC-*GBil* and Col-NLC-*GBil*-GTE (24 h) could increase the apoptosis intensity, e.g., 47.3% and 45% apoptosis, respectively, as compared to Doxorubicin, which showed 28.5% apoptosis (Figure 5C).

In previous reports, both categories of phytochemicals, *GBil* and GTE, led to the release of cytochrome c from mitochondria and the activation of Caspases-3 and -7, leading to the cleavage of cellular substrates and tumor cell death. Caspases-3 and -7 are responsible for the induction of apoptosis by EGCG in human ovarian cancer [72]. In addition to these mechanisms, the inhibition of angiogenesis is also a hypothesis. Compounds in *Ginkgo biloba* may reduce the formation of new blood vessels that feed the tumor, by inhibiting pro-angiogenic factors, e.g., VEGF (vascular endothelial growth factor) and by promoting anti-angiogenic ones [17]. Also, the possibility that phytocompounds in GTE and *GBil* inhibit metastasis and affect the ability of cancer cells to migrate and invade neighboring tissues by modulating the expression of matrix metalloproteinases [73] should not be overlooked.

#### 2.3.3. Cell Cycle Arrest by Col-NLC-GBil, NLC- and Col-NLC-GBil-GTE

**Figure 6:**
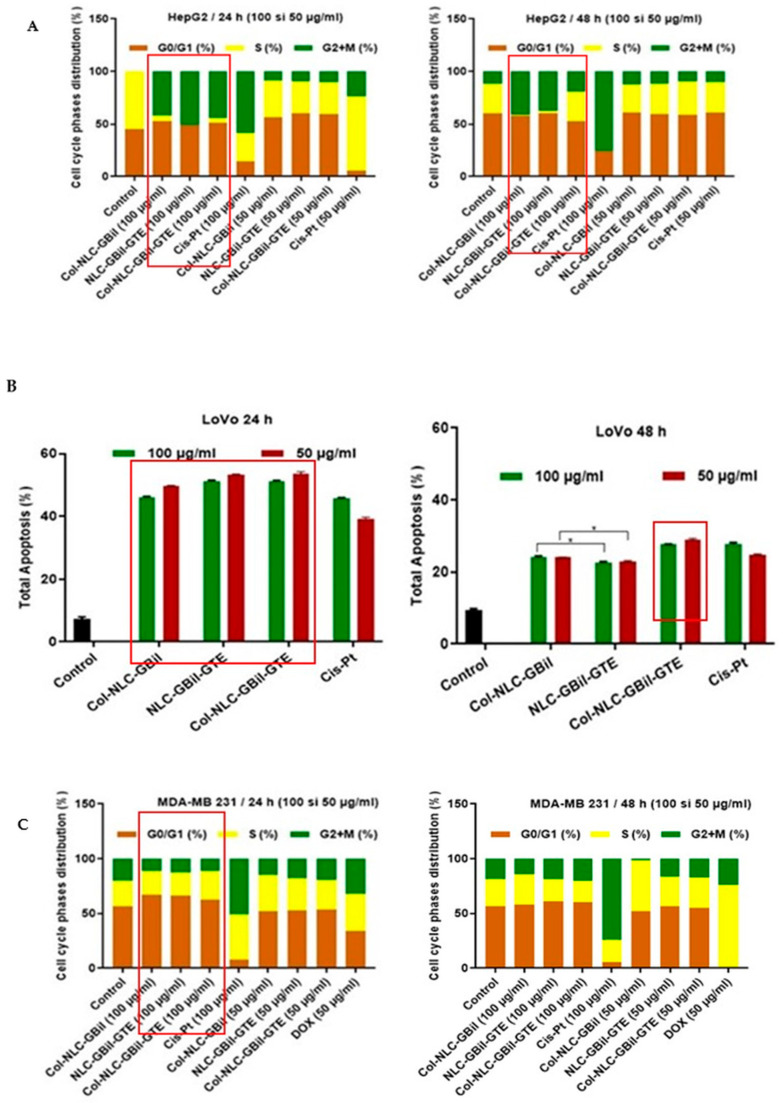
Cell cycle analysis (flow cytometry) performed on tumor lines: HepG2 (**A**), LoVo (**B**) and MDA-MB 231 (**C**).

To analyze whether the induction of cell cycle arrest contributed to the antiproliferative efficacy of NLC in cancer cells, cell cycle analysis was performed (Figure 6). In the majority, the treatment of cells with NLC and Col-NLC phytochemicals for 24 and 48 h caused a gradual increase in the cell population in the G0/G1 phase compared to synthetic drugs, DOX or Cis-Pt. Our results showed that bioactive GTE and *GBil* entrapped into an NLC can accompany the cell cycle progression of cancer cells, arresting their evolution at a certain phase and thus preventing uncontrolled tumor proliferation.

In the case of liver tumor cells, HepG2 (Figure 6A), an accumulation of hepatocyte cells in the G0/G1 phase was observed upon treatment with 100 mg/mL Col-NLC-*GBil*, NLC and Col-NLC-*GBil*-GTE, simultaneously with their decrease in the S phase, which tends to 0%. The induction by the NLC of an increase in cells in the G0/G1 phase may indicate a blockage of cell entry into the S phase, with a reduction in the number of cells entering the division cycle. Thus, the cells are “stopped” before DNA replication begins, limiting tumor proliferation.

Cell cycle arrest in the G1 phase induced by Epigallocatechin gallate (EGCG) from green tea extract has been demonstrated in various studies. According to several studies, the treatment of biliary cancer cells [74] and human colorectal cancer cells [75] with green tea extract or EGCG led to a significant increase in the population of cells in the G1 phase and a corresponding decrease in cells in the S phase. Catechin derivatives from GTE can activate the tumor suppressor protein p53, a protein with a defining role in the G1 phase. G1 cell cycle arrest may also be the result of the modulation of key proteins involved in the progression from G1 to S, such as Cyclin D1/E and Cyclin-dependent Kinases (CDKs) [76].

In addition to the antitumor contribution of GTE, phytocompounds from Ginkgo biloba can also contribute to the cell cycle progression of cancer cells, stopping them at a certain phase and thus preventing their uncontrolled proliferation. Like GTE, *GBil* acts by modulating the activity of key cell cycle regulatory proteins, such as Cyclins, CDKs and CDK inhibitors [77]. Outside this, the *Ginkgo biloba* extract also showed a significant increase in the population of cells in the G1 phase (indicating DNA fragmentation, specific for cell apoptosis) and G0/G1 cycle arrest in cancer cells [78].

Conversely, notable differences are also visible between the two concentrations of NLCs tested. For instance, at 100 mg/mL (24 h), increases in the LoVo cell population in the G2 + M phase were reported (Figure 6B). This may suggest an interference of NLCs with the processes preparatory to mitosis; as such, the cells are blocked before or during mitotic division. This cell cycle blockade is generally accompanied by the induction of apoptosis, a sign of the therapeutic efficacy of NLC.

Cell cycle distribution showed that NLCs with mixed phytochemicals (GTE + *GBil*) could arrest HepG2 and LoVo tumor cells in the G2 + M phase, which confirms previous apoptotic results. A prolonged block in G2 + M is often a signal for cells to trigger apoptosis. For example, in the case of LoVo cells, 100 mg/mL NLC- and Col-NLC-*GBil*-GTE caused the accumulation of cells in the G2 + M region, while Cis-Pt showed a higher cell accumulation in the G0/G1 phase compared to the control (Figure 6B, 24 h). These results suggested that NLCs had a beneficial cellular internalization and showed a higher toxicity than synthetic drugs, with an increasing cell population in the G2 + M phase. In previous research, phytocompounds from GTE and *GBil* have been shown to induce cell cycle arrest in the G2 + M phase and could modulate the p53 protein that prevents cells from entering the mitotic stage [79]. A study by Kim et al. showed that green tea polyphenol EGCG induces G2 + M cell cycle arrest and apoptosis in human lung cancer cells through p53-modulation [36]. In addition, phytocompounds from *GBil* can disrupt mitotic spindle formation or activate G2 + M checkpoints (e.g., by activating Chk1/Chk2), preventing mitosis entry or progression [38,80]. This may involve disrupting the process of mitosis or activating G2 + M checkpoints, which monitor DNA integrity and preparation for mitosis. As such, NLC-*GBil*-GTE could overexpress p53 and, at the same time, arrest the cell cycle in the G2 + M phase.

A notable interference of NLC- and Col-NLC-*GBil*-GTE is visualized by monitoring the changes in the population of cells in the S phase (DNA synthesis). Phytocompounds in GTE and *GBil* can induce DNA damage or directly interfere with DNA synthesis, impacting the population in the S phase. In almost all treatment groups with 100 mg/mL, the cell proportion in the S phase of the cell cycle decreased significantly, except for the MDA-MB 231 category (Figure 6C). A study by Shen et al. reported the induction of S phase arrest and apoptosis by EGCG in human hepatocellular carcinoma cells [81]. An accumulation of cells in the S phase may suggest a slowdown or arrest of DNA replication. This replicative stress may also culminate in apoptosis.

An effective antitumor activity of NLC-*GBil* and NLC-*GBil*-GTE was also determined in the case of colon cancer cells, LoVo, with NLCs acting by interfering with uncontrolled cell proliferation. The visualization of specific changes in the distribution of cell cycle phases, with the notable effect observed upon treatment with 100 mg/mL Col-NLC-*GBil* and Col-NLC-*GBil*-GTE, is shown in Figure 6B.

The influence of NLC phytochemicals and Col-NLC phytochemicals on breast cancer cells MDA-MB 231 was not so evident (Figure 6C). However, compared to the DOX drug, in the case of NLCs, there was an increase in the population in the G0/G1 phase. This suggests the effect of stopping cell division and, implicitly, a direct stop of entry into the S phase (blocking DNA replication and often inducing apoptosis).

## 3. Materials and Methods

### 3.1. Materials

Soy lecithin, Tween 20 (polyoxyethylene derivative of sorbitan monolaurate), Trolox (6-hydroxy-2,5,7,8-tetramethylchroman-2-carboxylic acid), potassium persulfate, 2,2-azinobis-(3-ethylbenzthiazoline-6-sulfonic acid) (ABTS), gallic acid, anhydrous sodium carbonate, NaCl and Folin–Ciocâlteu reagent were purchased from Sigma Aldrich Chemie GmbH (Munich, Germany). The sesame oil and cocoa butter were provided from S.C. Herbavit SRL (Oradea, Romania), and Glycerol monostearate (GMS) was acquired from Cognis GmbH (Monheim am Rhein, Germany). *Gingko biloba* extract (GBil) was provided by A.C. Helcor Pharmaceutical Co. (Baia-Mare, Romania), which was produced by Organic Herb Inc. (Changsha, China) and contained 24% Flavones (HPLC) and 6% Terpene Lactones (HPLC). The hydrolyzed collagen (Col) was purchased from the National Research and Development Institute for Textiles and Leather, Romania.

Doxorubicin (DOX), Cisplatin (Cis-diammineplatinum(II) dichloride, Cis-Pt), PBS/1 mM EDTA, L-Glutamine (Glu), Penicillin (100 units/mL), Streptomycin (100 μg/mL), Dulbecco’s modified Eagle’s medium (DMEM), fetal bovine serum (FBS), Propidium Iodide (PI) (stock solution 4 mg/mL PI in PBS) and RNase A (stock solution 10 mg/mL RNase A) were purchased from Sigma Aldrich (St. Louis, MO, USA). Annexin V-FITC kit and CycleTEST PLUS DNA Reagent kit were purchased from Becton Dickinson Biosciences, San Jose, CA, USA. The stock solutions were prepared by dissolving the compounds in a minimum amount of DMSO, kept at −20 °C. The working solutions were prepared from the stocks with the culture medium before each experiment.

### 3.2. Obtaining of NLC and Collagen-Decorated NLC Loaded with GBil and GTE

For the preparation of NLC and collagen-decorated NLC entrapping of *GBil*, GTE and *GBil* + GTE, the melt emulsification technique coupled with high-pressure homogenization was used as previously reported by the authors [82,83]. In outline, the lipid phase formed by cocoa butter (3.5 g), sesame oil (3 g) and glycerol monostearate (3.5 g) was combined (at 70 °C) with the aqueous phase containing the surfactant mixture (1.7 g Tween 20 and 0.3 g soy lecithin) and the phytochemical blend (1 g *GBil,* 0.2 g GTE and blend of *GBil* and GTE).

The formed pre-emulsion was maintained for 15 min at 70 °C under agitation. Afterwards, each pre-emulsion with a variable phytochemical mixture was submitted to HSH (High Shear Homogenizer PRO250, Oxford, CT, USA) at 12,000 rpm for 1 min. and HPH (APV 2000 Lab Homogenizer, Holzwickede, Germany) for six homogenization cycles at 400 bars, for 3.17 min. The obtained nanodispersions were cooled at room temperature, stored overnight at −25 °C and freeze-dried by lyophilization (−55 °C, 0.05 mbar, 54 h, using a Martin Christ Alpha 1-2 LD Freeze Dryer, Osterode am Harz, Germany). For decoration of NLC with hydrolyzed collagen, 20 mL of aqueous collagen solution 5 mg/mL was added dropwise, under vigorous stirring (for 3 min), in 40 mL of NLC dispersion (37 °C). By lyophilization of the aqueous dispersions of polymer–lipid nanocarriers, the formulations of Col-NLC-*GBil,* Col-NLC-GTE and Col-NLC-*GBil*-GTE were obtained, respectively.

### 3.3. Characterization of Developed NLC and Collagen-NLC Formulations

#### 3.3.1. Particle Size Evaluation

The mean hydrodynamic diameter (Z_ave_) and polydispersity index (PdI) of lipid nanocarriers containing *GBil*/GTE/GTE + *GBil* and subsequently decorated with hydrolysed collagen were determined by dynamic light scattering (DLS) using a Zetasizer Nano ZS analyzer (Malvern Instruments Ltd., Worcestershire, UK). The NLC–aqueous dispersions (diluted with water for an adequate scattering intensity) were measured at 25° and a 90° scattering angle. The intensity distribution was used to assess the particle size data. The average of three distinct measurements was used to calculate each Zave and PdI value. Through the DLS technique, the Brownian motion of suspended particles was statistically analyzed, and the obtained diffusion coefficient was transformed into size, using the Stokes–Einstein equation:

(1) $$D=\frac{kT}{6\pi \eta R}$$

where D = diffusion coefficient (m^2^/s); k = Boltzmann constant (J/K); T = temperature (K); η = dynamic viscosity of the liquid (Pa s); and R = hydrodynamic radius of the particle (nm).

#### 3.3.2. Determination of Zeta Potential

The surface charges of developed NLCs were measured using the electrophoretic light scattering method (Zetasizer Nano ZS, Malvern Instruments Inc., Worcestershire, UK). The zeta potential (ξ) was measured in a capillary cell (using the Helmholtz–Smoluchowski equation) by converting the particle electrophoretic mobility. Prior to ξ determination, aqueous NLC and Col-NLC dispersions were corrected to a conductivity of 50 μS/cm with 9% NaCl. Every measurement was made three times.

#### 3.3.3. Entrapment Efficacy of GBil and Green Tea Extract

The entrapment efficiency was determined by determining the quantity of polyphenols extracted in water from a known quantity of lyophilized NLC and Col-NLC phytochemicals. The polyphenolic content, expressed as gallic acid equivalents (GAE), was determined by using the Folin–Ciocâlteu method (ISO 14502-1:2005). Each suspension of 0.15 g lyophilized NLC- and Col-*GBil*/NLC-GTE/ NLC-*GBil*-GTE with 1 mL of water was gently shaken and subjected to 15,000 rpm (5 min), followed by the sampling of 0.5 mL of supernatant. The supernatant was mixed with 0.5 mL Folin–Ciocâlteu reagent 10% (*v*/*v*) and 4 mL solution Na_2_CO_3_ 7.5%. The mixture was allowed to react for 1 h at room temperature in a dark place, and then the absorbance was recorded at λ = 765 nm in triplicate, by using a UV-Vis spectrophotometer V670 Jasco (Tokyo, Japan). A calibration curve of gallic acid solutions was constructed (with R^2^ = 0.9919, 0–100 mg/L gallic acid). The entrapment efficiency of *GBil*/GTE and the blend of *GBil* and GTE was calculated using the following equation:

(2) $$EE\left(\%\right)=\frac{C_{a}}{C_{e}}\cdot 100$$

where *C_a_* = polyphenol content in the NLC and *C_e_* = polyphenol content in the *GBil*/GTE/*GBil* + GTE.

#### 3.3.4. In Vitro Determination of Antioxidant Activity

The in vitro antioxidant activity was determined according to the ABTS assay. The cationic radicals ABTS^+^ were generated by the reaction between 7 mM ABTS solution and 2.45 mM potassium persulfate solution (16 h, dark conditions, 4 °C). The ABTS^+^ solution was normalized for the absorbance of 0.700 (±0.01) at 734 nm (ABTS^+^). Each solution prepared by reaction between 2 mL NLC solution (5 mg/mL) and 3 mL of ABTS^+^ solution was subjected to spectrophotometric analysis. The absorbance of the samples (analyzed in triplicate) was measured after 4 min using ethanol as a reference. The average of the results was used for calculating the percentage of inhibition according to the following equation:

(3) $$\%\;inhibition=\frac{A_{0}-A_{s}}{A_{0}}∙100$$

where A_0_ = the absorbance of blank (3 mL ABTS^+^ and 2 mL ethanol) and A_s_ = the absorbance of the sample.

The IC_50_ value (defined as the concentration at which 50% of ABTS^+^ is scavenged) was determined by varying the NLC concentrations in the range 0.05–2 mg/mL.

#### 3.3.5. Cell Cultures and Treatments

Human hepatic adenocarcinoma (HepG2), human colorectal adenocarcinoma (LoVo), human breast adenocarcinoma (MDA-MB-231) and human umbilical vein endothelial cells (HUVEC) were purchased from the American Type Culture Collection (ATCC, Manassas, VA, US). HUVEC normal cells, isolated from the vascular endothelium of an umbilical cord, were used as a reference. Adherent cells were maintained in culture in Dulbecco’s modified Eagle medium/nutrient mixture F-12 (DMEM: F12) with 2 mM of L-glutamine, 10% fetal bovine serum, 100 units/mL of penicillin and 100 µg/mL of streptomycin and incubated at 37 °C in a 5% CO2-humidified atmosphere. After 24 h, when the cells reached approximately 60% confluence, adherent cells were treated with varying concentrations of the compounds for different periods. Cis-Pt and DOX, conventional oncology drugs used in cancer treatments, can be used as a positive control for the experiments. Cell treatments of compounds, Cis-Pt and DOX, were carried out using concentrations of 400, 200, 100, 50, 25, 12.5, 6.25 and 3.125 µg/mL of the tested compound. Then, cells from the flasks were detached using a non-enzymatic solution of PBS/1 mM EDTA, washed twice with PBS, and used for proliferation/cytotoxicity assays or evaluation of apoptotic events using flow cytometry. Alternatively, cells were fixed in ice-cold ethanol/PBS (70:30) and kept until use at 4 °C for cell cycle analysis using the flow cytometry technique. In all experiments described in this study, all untreated cells were designated as control cells [84].

#### 3.3.6. Assessment of Compounds’ Cytotoxicity by MTS Assay

The cytotoxic potential of the tested compounds was evaluated in comparison with two standard chemotherapeutic agents, cisplatin (Cis-Pt) and doxorubicin (DOX), which served as positive controls. Cancer and normal cells (1 × 10^4^ cells/well) were seeded in 96-well plates (100 µL/well) and cultured for 24 h. After removing the culture supernatants, cells were treated for either 24 h or 48 h with increasing concentrations of the test compounds or reference drugs. Cytotoxicity was assessed using the CellTiter 96^®^ Aqueous One Solution Cell Proliferation Assay (Promega, Madison, WI, USA). This assay contains the tetrazolium compound MTS (3-(4,5-dimethylthiazol-2-yl)-5-(3-carboxymethoxyphenyl)-2-(4-sulfophenyl)-2H-tetrazolium, inner salt) and the electron coupling reagent PES (phenazine methosulfate), which together enable metabolically active cells to reduce MTS into a soluble colored formazan product. Following treatment, 20 µL of MTS/PES reagent was added to each well, and the plates were incubated with gentle agitation for 4 h at 37 °C. The resulting color intensity, proportional to the number of viable cells, was measured spectrophotometrically at λ = 492 nm using a Dynex ELISA reader (DYNEX Technologies-MRS, Chantilly, VA, USA). Cell viability (%) was calculated using the following formula:

Cell viability (%) = (T − B)/(U − B) × 100(4)

where T = absorbance of treated cells, U = absorbance of untreated control cells (considered 100% viable) and B = absorbance of the blank (medium only)

All results are presented as mean ± standard deviation (SD) of three independent experiments (*n* = 3).

#### 3.3.7. Apoptosis Analysis by Flow Cytometry

The analysis was performed with the Annexin V-FITC Apoptosis Detection Kit (BD Biosciences, San Jose, CA, USA) following the manufacturer’s protocol. Unstained cells were included as negative controls. Tumor and normal cells were first cultured for 24 h in complete medium before being treated for an additional 24 h with the tested compounds. After the treatment, cells were detached using PBS containing 1 mM EDTA, washed twice with PBS and centrifuged at 300× *g* for 5 min. The cell pellets were then resuspended in 400 µL of binding buffer, and 100 µL aliquots were transferred into flow cytometry tubes for staining. Each sample was incubated with 5 µL of Annexin V-FITC and/or 5 µL of Propidium Iodide (PI) for 15 min at room temperature in the dark. Untreated (NT) cells served as controls. Apoptotic events were quantified using either a FACScan or FACS Canto II cytometer (BD Immunocytometry Systems, Mountain View, CA, USA). Data acquisition and analysis were conducted with DIVA 6.2 software, which enabled the discrimination of the following: early apoptosis (Annexin V^+^/PI^−^), late apoptosis (Annexin V^+^/PI^+^) and necrosis (Annexin V^−^/PI^+^) [85].

#### 3.3.8. Cell Cycle Evaluation Using Flow Cytometry

We analyzed the DNA content of tumoral cells by flow cytometry approaches to demonstrate the potential modulation of the cell cycle phases following the treatments with the compounds studied. Before cell cycle analysis, ethanol-fixed cells (1 × 10^6^) were washed twice in cold PBS, and 0.5 mL of the FxCycle^TM^PI/RNase Staining Solution was added, a PI staining solution that includes DNase-free RNase A and a permeabilization reagent. The cells were resuspended and incubated at room temperature (RT) for 10–30 min in the dark. Then, 3 × 10^4^ events were acquired through the flow cytometry technique using a FACS CANTO II flow-cytometer (Becton Dickinson-BD, Immunocytometry System, Mountain View, San Jose, CA, USA), and analyses were performed using the DIVA 6.2 software [86].

#### 3.3.9. Statistical Analysis

All measurements were performed in triplicates. All data analyses were performed using GraphPad Prism 7 (GraphPad Software Inc., La Jolla, CA, USA). The differences between the treatment and control groups or between different treatments were statistically analyzed using one-way analysis of variance ANOVA. Statistical significance was considered at *p* < 0.05.

## 4. Conclusions

This approach provides insightful information regarding the development of dual-captured *Ginkgo Biloba* and green tea extract in Collagen-Nanostructured Lipid Nanocarriers (Col-NLC-*GBil*-GTE), which could display ideal synergistic cytotoxic effects against HepG2 hepatocytes and LoVo colon cancer cells. NLC and collagen-decorated NLC containing a phytochemical mixture were characterized in terms of particle size, physical stability, encapsulation efficiency and in vitro biological activity. The NLC size was <190 nm, and the polydispersity index values were <0.19, which suggests the obtaining of homogeneous delivery systems with a relatively monodisperse population. The colloidal nanocarriers had a negative surface charge, with zeta potential values more electronegative than −40 mV, which can prevent colloidal aggregation and flocculation over time.

A synergistic effect was observed between *GBil* and GTE regarding their stability and biological efficiency, and collagen provided an improvement in biocompatibility, although it slightly reduced the encapsulation efficiency and antioxidant activity. NLC formulations containing GTE showed a slightly higher entrapment efficiency than those containing only *GBil,* which may be correlated with a better structural and conformational adaptability of GTE in the nanocarrier matrix. The decoration of NLC-*GBil* with hydrolyzed protein insignificantly decreases the entrapment efficiency; for instance, EE% = 85.17 ± 0.67 for NLC-*GBil*-GTE versus 81.91 ± 1.37 for Col-NLC-*GBil*-GTE, respectively.

NLC phytochemicals successfully fulfilled their role as strong antioxidants. The most effective was found to be the NLC containing GTE, which showed an excellent capacity to scavenge the cationic radicals, with a maximum inhibition of 97.9% ± 0.43 and an IC_50_ value of 0.08 mg/mL, which supports its potential in preventing cellular oxidative stress. The antioxidant activity of NLCs was slowly reduced when they were decorated with collagen because of the interactions between the phytocompounds from GTE and *GBil* and collagen.

The in vitro evaluation of the cytotoxic potential on cancer cell viability (over 24 h treatment) showed a <20% decrease in the viability of colon tumor LoVo cells in the presence of Col-NLC-*GBil*-GTE, which may be related to the successful Col-NLC cellular internalization. The effect of NLC-herbal principles and Col-NLC-herbal principles on normal HUVECs showed a lack of cytotoxicity, with the cell viability being higher than 80% at concentrations ≤ 100 mg/mL.

The apoptosis outcomes of NLC and Col-NLC underlined a slightly more pronounced efficacy of the NLC- and Col-NLC-phytochemical mixes compared to the usual drug, Cisplatin. The 50 mg/mL NLC- and Col-NLC-GBil-GTE appeared to significantly induce a 50% greater apoptosis after its addition to LoVo cancer cells compared with the control group (for 24 h); for instance, NLC-*GBil*-GTE induced 53.2% apoptosis, Col-NLC-*GBil*-GTE induced 53.5% apoptosis and 50 μg/mL Cis-Pt resulted in 39.3% apoptosis. Moreover, a 20% increase in apoptosis for HepG2 cells was registered for a treatment with 100 μg/mL NLC-GBil-GTE compared to Cis-Pt, with 63.4% total apoptosis for NLC-*GBil*-GTE and 59% for Col-NLC-*GBil*-GTE, while the total apoptosis induced by 100 μg/mL of a chemotherapeutic drug, Cis-Pt, was 52.6%.

GTE and *GBil* entrapped in an NLC arrested the cell cycle progression of cancer cells at a certain phase, thus preventing uncontrolled tumor proliferation. An accumulation of hepatocyte HepG2 cells in the G0/G1 phase was observed upon treatment with 100 mg/mL NLC- and Col-NLC-*GBil*-GTE, simultaneously with a decrease in the S phase. Phytocompounds from GTE and *GBil* can induce DNA damage or directly interfere with DNA synthesis, impacting the population in the S phase. These results support the idea that NLC accompanies a blockage of cell entry into the S phase, which results in a reduction in the number of cells that could enter the division cycle.

In addition, cell cycle distribution showed that NLCs with mixed phytochemicals could arrest HepG2 and LoVo tumor cells in the G2 + M phase, which confirms previous apoptotic results. Our results showed that the treatment of cells with NLCs led to cell cycle arrest in the G2 + M phase, thereby reducing the proportion of cells in the G0-G1 phase. The G2 + M arrest of tumor cells was significant in cells treated with NLC-*GBil*-GTE compared to all other treatment groups. In almost all treatment groups, the proportion of cells in the S phase of the cell cycle was significantly reduced. An increase in the percentage of cells in the G1 phase and a corresponding decrease in cells in the S and G2/M phases indicated a blockage of entry into the DNA synthesis phase.

Thus, it may be concluded that the developed Col-NLC phytochemical gained the ability of the co-delivery of *GBil* and GTE to increase hepatocyte and colon cancer therapeutic efficacy in vitro. A connection of Col-NLC with the GBil extract alongside the main chemotherapeutic polyphenols (from GTE) could augment the sensitization of cancer cells to phytochemicals or conventional drugs, is able to concurrently reduce the side effects of synthetic drugs and may lead to a promising increase in therapeutic efficacy. Our findings may open a horizon for complete in vivo studies and further investigation in future antitumor therapeutic strategies.

## Figures and Tables

**Table 1:** Entrapment efficiency of *GBil* and GTE into NLC and collagen-decorated NLC.

NLC and Collagen-Decorated NLC	EE%
NLC-GBil	72.30 ± 0.74
Col-NLC-GBil	71.39 ± 1.76
NLC-GTE	86.75 ± 1.21
Col-NLC-GTE	83.59 ± 0.62
NLC-GBil-GTE	85.17 ± 0.67
Col-NLC-GBil-GTE	81.91 ± 1.37

## Data Availability

The raw data supporting the conclusions of this article will be made available by the authors upon request.
